# Probing the Mechanical Properties of Porous Nanoshells by Nanoindentation

**DOI:** 10.3390/nano12122000

**Published:** 2022-06-10

**Authors:** Felipe J. Valencia, Viviana Aurora, Max Ramírez, Carlos J. Ruestes, Alejandro Prada, Alejandro Varas, José Rogan

**Affiliations:** 1 Departamento de Computación e Industrias, Facultad de Ciencias de la Ingeniería, Universidad Católica del Maule, Talca 3480112, Chile; aprada@ucm.cl; 2 Centro para el Desarrollo de la Nanociencia y la Nanotecnología, CEDENNA, Avda. Ecuador 3493, Santiago 9170124, Chile; max.ramirez@gmail.com (M.R.); alejandro.varas@gmail.com (A.V.); jrogan@uchile.cl (J.R.); 3 Departamento de Física, Facultad de Ciencias, Universidad de Chile, Casilla 653, Santiago 7800024, Chile; viviana.aurora@gmail.com; 4 Instituto Interdisciplinario de Ciencias Básicas, CONICET-UNCuyo, Facultad de Ciencias Exactas y Naturales, Universidad Nacional de Cuyo, Mendoza 5500, Argentina; cjruestes@hotmail.com

**Keywords:** porous nanoshells, molecular dynamics, porous materials, nanoindentation, plasticity

## Abstract

In this contribution, we present a study of the mechanical properties of porous nanoshells measured with a nanoindentation technique. Porous nanoshells with hollow designs can present attractive mechanical properties, as observed in hollow nanoshells, but coupled with the unique mechanical behavior of porous materials. Porous nanoshells display mechanical properties that are dependent on shell porosity. Our results show that, under smaller porosity values, deformation is closely related to the one observed for polycrystalline and single-crystalline nanoshells involving dislocation activity. When porosity in the nanoparticle is increased, plastic deformation was mediated by grain boundary sliding instead of dislocation activity. Additionally, porosity suppresses dislocation activity and decreases nanoparticle strength, but allows for significant strain hardening under strains as high as 0.4. On the other hand, Young’s modulus decreases with the increase in nanoshell porosity, in agreement with the established theories of porous materials. However, we found no quantitative agreement between conventional models applied to obtain the Young’s modulus of porous materials.

## 1. Introduction

Porous nanoparticles have demonstrated superior capabilities in, for example, catalysis, plasmonics, sensing, energy storage [1,2,3,4]. Despite being a less studied field, the mechanical behavior of nanoparticles has been a worthy subject of study in the last years due to their unexpected mechanical properties [5,6,7,8,9,10]. Mechanical properties in nanoparticles can be advantageously tailored through several factors such as nanoparticle size [11,12], lattice orientation [13], and surface engineering [14,15,16,17]. All those factors directly impact the elastic and plastic behavior of the nanostructures.

Recent studies have addressed the role of porosity on nanoparticles. An example is an improvement in the plasticity of carbon nanoparticles with porous structures [18]. In the same direction, hollow nanoparticles can considerably enhance the strain required to drive the nanostructure fracture, which is an advance considering the brittle nature of carbon materials [19]. Using molecular dynamics simulations, Valencia et al. [20], and Yang et al. [6] showed that porous nanoshells (NS) display an excellent combination of strength and flexibility, which can be controlled at the same time by adjusting the size of the cavity. These studies contemplate only single-crystal and defect-free NS, which are difficult to obtain with current experimental techniques [21,22,23]. An approximation closer to experiments was conducted by considering the mechanical properties of polycrystalline shells with different grain sizes [24]. As a result, polycrystalline hollow NS can show yield stress depending on the grain size, following reverse Hall–Petch dependence supporting the statement of Shan et al. [5] about the ultrahigh strength on Cd–Se hollow nanospheres due to the hierarchical design of those nanostructures.

Besides the case of polycrystalline NS, the invasive nature of some synthesis processes results in the synthesis of hollow NS with porous structures [25,26,27]. Porous NS have shown an advance with their nonporous counterparts regarding catalysis, sensing, and storage [28,29,30]. The high performance of porous NS is due to their enhanced surface-to-volume ratio over their nonporous counterparts. Due to their convenient mechanical properties, porous materials have fueled numerous studies to unveil their mechanical properties in almost every kind of material [31,32,33,34,35]. In this aspect, porous NS with hollow designs can present the attractive mechanical properties as observed in hollow NS, but coupled with the unique mechanical behavior of porous materials. This last point is precisely the focus of this contribution.

In particular, the mechanical behavior of Au-based porous NS is studied as a model structure for face-centered cubic (fcc) materials. The choice of the studied material is motivated by several factors: (i) there are many experiments that have synthesized Au porous NS with a size of a few to tens of nanometers; [36,37] (ii) the large number of studies addressing the mechanical behavior of Au nanoporous materials; [32,38,39,40] (iii) the importance of Au hollow nanoparticles in fields such as catalysis, sensing, or plasmonics, where mechanical strain could be an interesting tool to modify the material performance [41,42].

## 2. Methods

Nanoindentation tests were performed through classical molecular dynamics simulations using LAMMPS code [43]. Hollow-porous nanostructures typically achieve sizes from tens up to a few hundred nanometers. Here, we simulate a porous NS with a size of 50 nm diameter and 5 nm of thickness in order to perform MD simulations in closeness with sizes of already synthesized Au nanoparticles [44,45,46,47]. We built the nanostructure following the same procedure as that of Valencia et al. [48] for porous NS. The NS used in our simulation was constructed from a polycrystalline Au sample (with an average grain size of 10 nm) by scabbing two concentric spheres with different radii. The nanocrystalline sample was obtained employing a Voronoi tessellation algorithm, following standard methods that had been described in the literature [49,50,51].

We first built the polycrystalline structure; then, we randomly removed some grains to give the sample some porosity. Therefore, we define NS porosity as follows:

$$\phi =1-\frac{V_{\mathrm{porous}}}{V_{\mathrm{hollow}}}\;,$$

where $V_{\mathrm{porous}}$ is the solid volume of the porous NS, and $V_{\mathrm{hollow}}$ is the solid volume of the hollow NS without porosity. To investigate the role of porosity, we relaxed four porous NS with ϕ = 0.126, 0.208, 0.306, and 0.441. ϕ=0.441 was chosen since porous hollow nanoshells with larger porosities are thermally unstable at room temperature [48], collapsing on solid nanoparticles. On the other hand, ϕ=0.126 corresponds to porous nanoshells with 6 pores, so that decreasing the value of phi did not contribute with additional physics than that observed for the pc case.

The porous NS constructed by our procedure could be out of equilibrium by several factors. On the one hand, excess energy due to the grain boundary structure can trigger the grain boundary sliding and the shrinkage of the NS [48]. On the other hand, while typical Au hollow nanospheres with thicknesses of 5 nm and diameters of 50 nm are stable at room temperature [52,53,54], the porous structure of the NS increases the surface area, which can drive the collapse of the nanostructure due to surface stresses [48]. Therefore, we adopted a relaxation time of 10 ns using a timestep of 1.0 fs at 400 K. Then, the system temperature was cooled to 300 K, in 0.1 ps with a time step of 1.0 fs. The temperature throughout the relaxation was controlled with a velocity rescale algorithm, and the atoms are integrated using the Microcanonical (NVE) ensemble.

There are several interatomic potentials available in the literature to model Au-based nanostructures. On the basis of the available literature on modeling Au nanofoams [55], we chose the embedded atom method [56] potential of Johnson [57] since this potential successfully reproduced physical parameters as elastic constants, surface energies, stacking fault (SF) energies, and point defects energies that are fundamental for the understanding of the mechanical properties of nanoporous materials.

To model the NS nanoindentation, we used the methodology of Yang et al. [6], which considers two identical infinite planes as indenters compressing the nanosphere at a given indentation velocity through a purely repulsive potential given by:

$$V=k{\left(z-z_{0}\right)}^{3}\;,$$

where $z_{0}$ defines the indenter surface, *z* is the *z*-position of the atoms, and k=10 eV/Å is a constant that describes indenter stiffness. We compressed the porous NS by displacing each indenter with a speed of 1.0 m/s. To obtain the contact pressure and hardness (*H*), we assumed an atomic contact area using the criteria of Ziengenhain et al. [58]

$$A=\sum_{N}A_{\mathrm{atom}}\;,$$

where $A_{\mathrm{atom}}$ is the atomic area, and *N* is the number of atoms in contact with the indenter. An atom was assumed in contact with the indenter if $|z-z_{0}|<0.2$ Å. In this way, hardness was obtained averaging the contact pressure for a strain interval of 0.15 to 0.40, as follows:

$$H=\frac{F}{A}\;.$$

During the whole nanoindentation process, the system temperature was maintained at 300 K, using a velocity rescale algorithm and a 1.0 fs time step. System visualization, rendering, and postprocessing were performed with the Open Visualization Tool (OVITO) [59]. Dislocations were inspected using the automated algorithm for the identification of lattice defects, Crystal Analysis Tool (CAT), the common neighbor analysis method (CNA), the polyhedral template matching method [60] (PTM), and the Dislocation eXtraction Algorithm (DXA) [61]. To perform an automated classification of complex defects such as the stacking faults or twins, we used the CAT algorithm, which performs an automated search of crystalline defects, associates a fingerprint to all the structures in the sample, and uses a series of descriptors to compare the defective structures with their internal database.

## 3. Results

**Figure 1:**
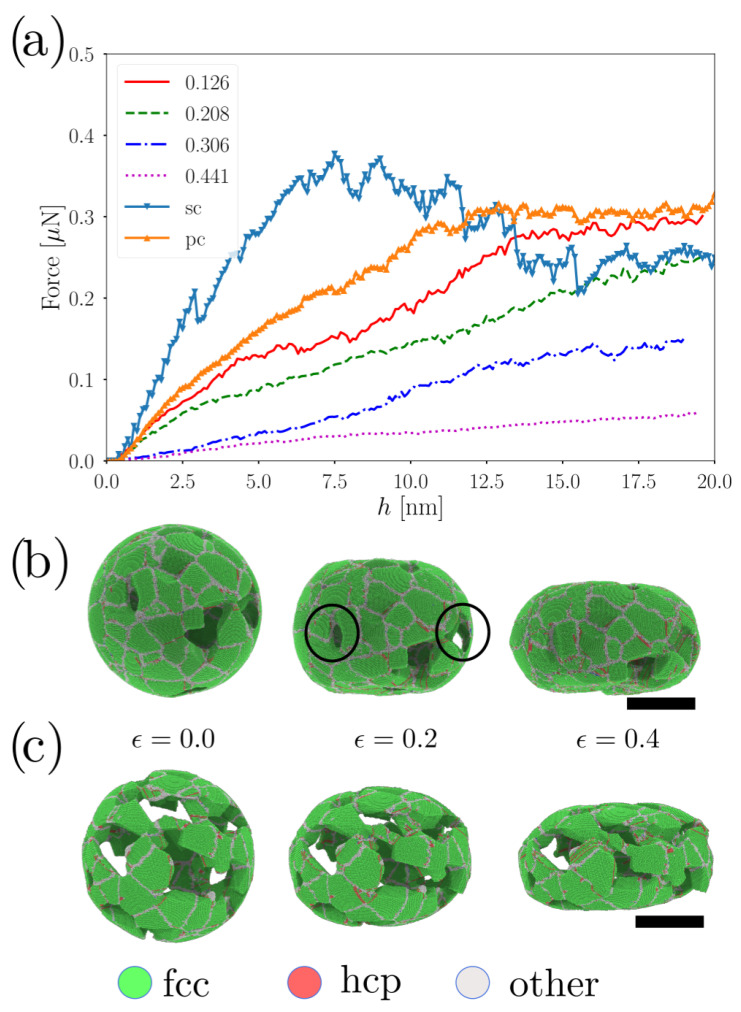
(**a**) Force vs. depth curves for different porous NS. (**b**,**c**) CNA at different strains for ϕ=0.208 and ϕ=0.441, respectively. Circles, color legend of CNA. Scale bars correspond to 15 nm.

The load–depth curves of the nanoindentation with flat indenters are presented in Figure 1a. The curves show that, under depth-controlled nanoindentation, the force continuously increased with depth but decreased with NS porosity. For nanoindentation simulations, *h* is defined as the distance below $z_{0}$, with $z_{0}$ the initial nanoparticle height. For all values of ϕ, there was an increase in the load as a function of *h*; however, for ϕ=0.441, the behavior was quite different. For such an extreme porosity value, force almost monotonically increased with indentation depth, suggesting a different plastic deformation mechanism compared to ϕ=0.126 and ϕ=0.208 cases. On the other hand, ϕ=0.306 showed mixed behavior from a monotonic increase for depths smaller than 10 nm to a sawlike increase for h>10 nm. To establish a comparison point in the behavior of the porous NS, we also plotted the curves for a single crystalline (sc) hollow nanoshell and a polycrystalline (pc) hollow nanoshell. Since both ps and sc do not present porosity, the force achieved by the indenter was higher than that for the porous NS. As expected, in the absence of defects, the sc NS achieved higher values of indenter force; however, for depths larger than 7.5 nm, indenter force decreased and behaved similarly to that of a NS with ϕ=0.306. The decrease in the strength of the sc NS was expected due to the nucleation of multiple partial dislocations traveling through the nanoparticle [20]. On the other hand, from the indentation curve for the pc NS, there was an overlap on the force–depth curve with the ϕ = 0.126 case for depths smaller than 1.5 nm. If we consider a Hertz contact model to describe the first stage of the nanoindentation process, both nanoparticles showed a Young’s modulus of E= 10 GPa. Beyond the elastic limit, we observed that the two curves differentiated mainly due to the porous nature of the NS with ϕ=0.126, which presents an onset of plasticity at lower loads. In the limit of h>15.0 nm the load curve for ϕ=0.126 and pc NS, converge to the same values. We can understand this last result because the high deformation field over the ϕ=0.126 caused the annihilation of some pores, forming a structure which is more closely related to a pc NS than a porous NS. In pc materials, dislocations are nucleated, travel and encounter grain boundaries or other dislocations to increase the material strength, in agreement to the Hall–Petch effect. Here, the effect was the opposite since the grains were smaller than 10 nm, favoring a reverse Hall–Petch effect, as was reported for Pd pc nanoshells [24].

Complementarily, Figure 1b,c show the deformation process for two NS with different porosities. CNA revealed that several nanoscale processes occurred as a function of the indenter depth. For instance, ϕ=0.126 showed the localization of hexagonal close-packed (hcp) planes in the neighbor of the NS pores (represented by red atoms in the figure), followed by homogeneous nucleation of partial dislocations and SF at strains larger than 0.2. The ϕ=0.126 case also showed the compaction of some pores (enclosed in black circles) at strains close to 0.4, to form a pc-like nanoshell. This transition from porous to pc nanoshell was consistent with that observed on the force–depth curve for larger indentation depths. For ϕ=0.441, SF faults do not increase dramatically from strain ϵ=0.2 to ϵ=0.4, suggesting that a different mechanism could mediate plastic deformation, such as grain boundary sliding.

**Figure 2:**
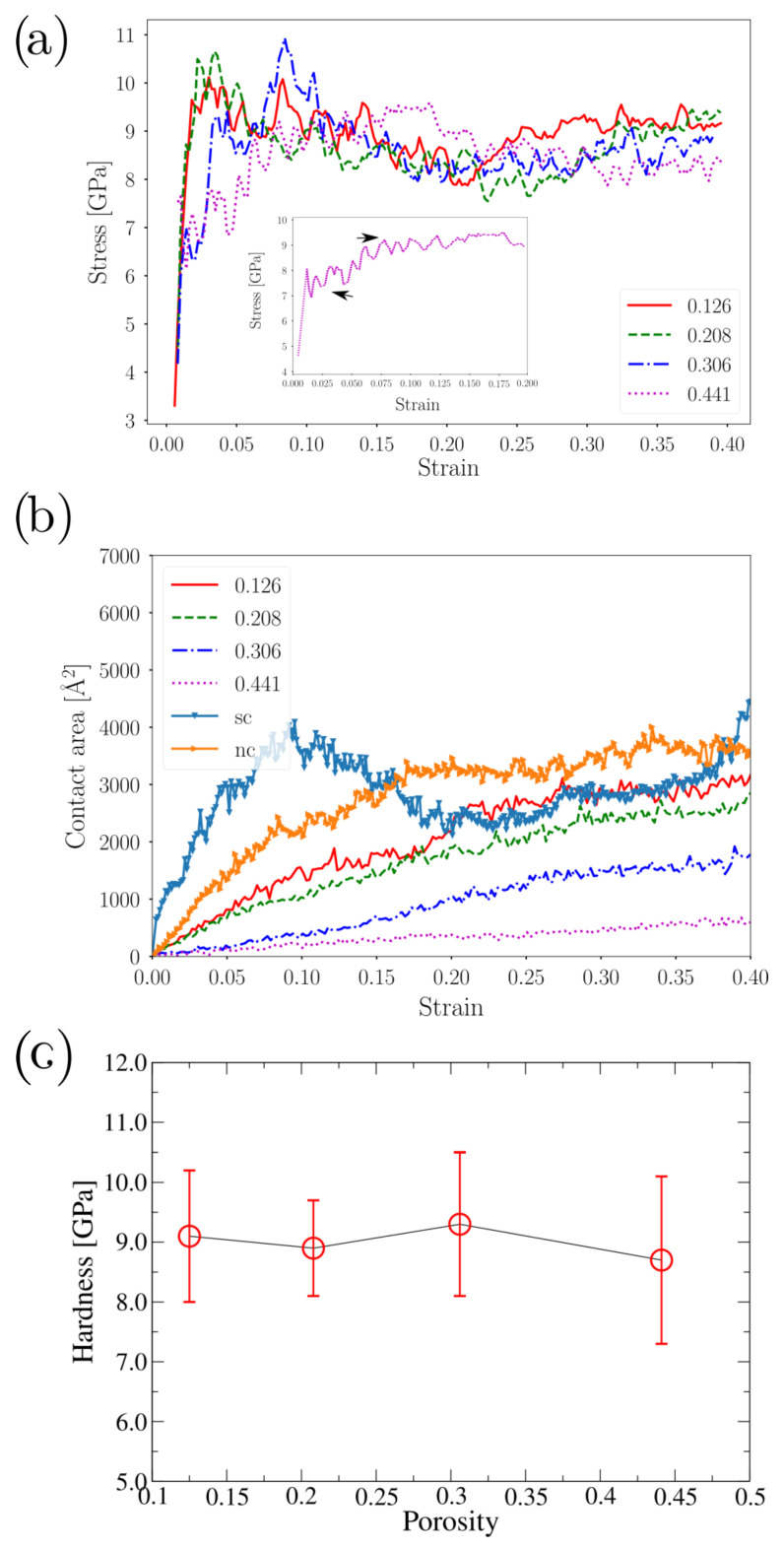
(**a**) Contact pressure curve. (inset) Magnification of contact pressure for ϕ=0.441 case, where arrows indicate a “plateau” on the contact pressure curve. (**b**) Atomic contact area as a strain function. (**c**) Hardness as a function of the NS porosity.

The indenter contact pressure tended to be almost the same for each indented NS, in contrast to the differences in the load-force curve. Figure 2a shows that contact pressure grew rapidly from 0 to 10 GPa for strains smaller than 0.03, leading to reasonably constant flow stress up to strains of 0.4. Quite different behavior was observed for ϕ=0.441, where two-step “hardening” was observed (inset Figure 2a. This behavior could be attributed to the higher porosity, which reduced the contact pressure in the first stage due to load-bearing network effects. Load-bearing network effects play a major role in other highly porous systems, such as nanoporous gold with low relative density.

We also observed differences in the force magnitude on the load vs. depth curve, which could be understood in terms of contact area. Figure 2b shows that the atomic contact area changed with porosity for all cases. The observed trends can be extrapolated to load curves, suggesting that the differences in force were mainly attributed to the contact area, besides any other size effect due to the NS porosity. The contact area significantly increased with the strain for sc NS, since the deformation mode of the sc NS was completely different from that of shells with polycrystalline structure. This occurs because plastic deformation is assisted by partial dislocation that travels for the entire nanoparticle. The stacking faults led by the initial partial dislocations, helps the structure to glide the SF direction favoring a transition from spherical to ellipsoidal nanoshell (for higher strains) increasing the contact area with the indenter [20]. This effect was reported for conventional nanoparticles, hollow nanoshells, and even metallic nanotubes.

**Figure 3:**
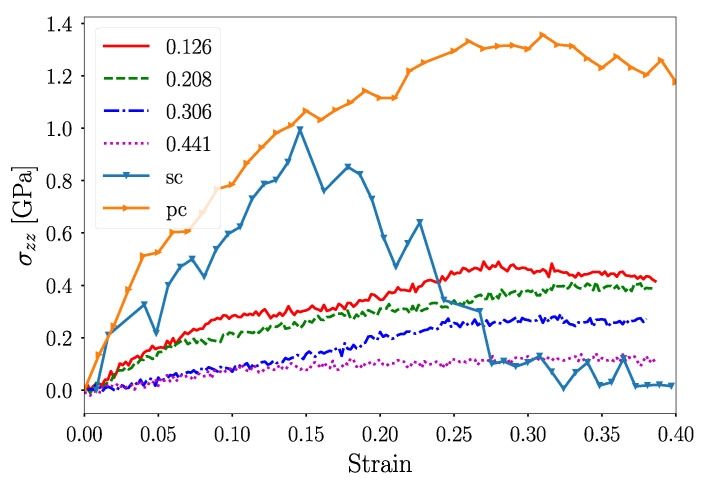
$\sigma_{zz}$ vs. effective strain.

As an alternative description, we could also calculate the NS internal stress $\sigma_{zz}$, as in Figure 3. We divided the $\sigma_{zz}$ into two groups. For ϕ≤0.208, the nanoparticles presented a stiffer response with similar values of the effective Young’s modulus. In contrast, for ϕ=0.306, ther was softer but ductile-like behavior. Reported values show stress in line with the uniaxial compression of porous gold nanofoams [55]. Only surface atoms were tested in contact pressure analysis. Instead, we averaged the $\sigma_{zz}$ value over the whole nanosphere describing the total stress of the nanostructure under compression. This alternative treatment gives a more detailed description of yield stress, as in the case of isotropic/periodic materials. Both pc and sc NS showed higher values than that observed for porous NS. The sc NS showed a high stress peak but it decreased abruptly after the onset of plasticity. Contrary to this, the pc NS showed much higher stress with a curve that continuously increased for deformations as high as 20 nm.

The hardness values obtained for our porous NS nanofoams were in the range of 8.5 to 9.0 GPa (Figure 2c), which were larger than those reported for Au nanofoams [38] (6 GPa). A possible explanation is that, in our case, we considered flat indenters instead of spherical tips, which could decrease the probability to produce plastic deformation. In order to relate contact pressure values with yield stress, we used an equation of the form of $\sigma_{0}=CH$, with hardness *H* defined from the atomic contact area and averaged in the strain interval of 0.2 to 0.4. Contact mechanics describe the hardness to yield stress relation between a flat surface and a solid sphere as [62]:

(1) $$\sigma =\frac{\left(1+2\nu \right)}{2}H\;,$$

with ν the material Poisson’s ratio. Assumming an average value of ν=0.4 [40], we obtain a constraint factor (C = 1.49) similar to the reported for microsized nanoparticles [62]. Equation (1) also renders a yield stress of 2.7 GPa, which is very close to the yield stress of Au nanowires under tension [63]. This deduction, also shows that the reasoning followed by Ruestes et al. [32] to determinate the relation between hardness and yield stress for nanoporous Au indented with spherical tips, can be also extended to porous nanospheres indented with flat punchs.

### 3.1. Elastic Regimen Calculations

**Figure 4:**
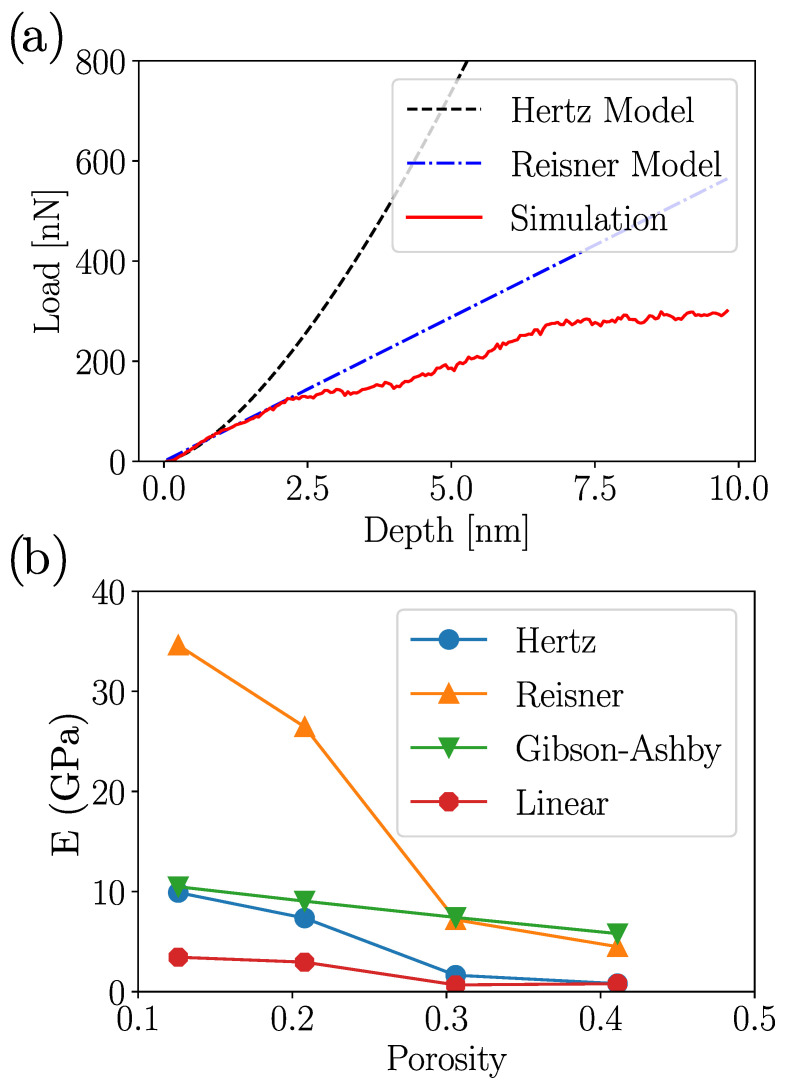
(**a**) Hertz, and Reissner fit from the load–depth curve for ϕ=0.128 case. (**b**) Comparison among Hertz, Gibson–Ashby, Reissner, and MD data for Young’s modulus. “Linear” corresponds to a linear fit of the stress–strain curve at strains smaller than 0.01. The Young’s modulus of Au bulk was 78 GPa.

To determine the Young’s modulus of the indented specimens, we can invoke elastic models for the curve fitting. A Hertz elastic model, which considers a solid nanosphere pushed by a rigid planar indenter, assumes a load *F*

$$P=\frac{4}{3}E_{r}\pi R^{3/2}h^{1/2},$$

where $E_{r}$ is the combined Young’s modulus, and *R* is the nanoparticle radii. The model predicts good agreement in the load force curve (Figure 4a), rendering $E_{r}$ values in the range 1–10 GPa in terms of the system porosity. A common trend, as expected, is the decrease in Young’s modulus *E*, with the increase of the NS porosity. Of course, the assumptions of a Hertz model do not consider any stress contribution from the void, somewhat giving larger values for *E*. On the other hand, the Reissner model [64,65], which introduces a shear stress component on the modeling of a thin shell deformed by flat indenters, describes that the indenter for force *F* can be modeled as:

$$F=\frac{4E_{0}w^{2}h}{R\sqrt{3(1-\nu^{2})}}\;,$$

where $E_{0}$ is the Young’s modulus of bulk Au, *w* is NS thickness, and ν is the material Poisson’s ratio. Figure 4a shows that Reissner model described the load vs. depth curve extremely well during the elastic deformation regime, suggesting that the inclusion of a shear stress contribution is fundamental to adequately describe the elastic response of uniaxially compressed NS.

In the same direction, reasonable *E* values could be extracted from a linear fit of the strain-stress curve (Figure 3) for strains smaller than 0.01. As observed in Figure 4b, the linear fits render values three times lower than those observed with the Hertz model. Alternatively, Gibson–Ashby theory describes the Young’s modulus as:

$$E=C_{0}E_{0}{\left(\frac{\rho }{\rho_{0}}\right)}^{3/2},$$

where ρ is porous material density, and $E_{0}$ and $\rho_{0}$ correspond to the Young’s modulus and density of the materials without porosity, respectively. The $C_{0}$ value was a constant that typically assumes a value of 0.65 for cellular materials. In our simulations, the porosity of the NS solely due to the cavity was 0.6. This value renders a relative density $\left(\rho /\rho_{0}\right)$ in the range of 0.3–0.4, which is suitable for the shell porosity. Assuming an elastic modulus of 78 GPa for bulk Au [66,67], the Gibson–Ashby model gives values close to the Hertz model in the limit of the lower shell porosities but in reasonable agreement with the effective Young’s modulus for the larger ϕ values.

### 3.2. Poisson’s Ratio Calculations

To better describe transverse elastoplastic deformations, we calculated the Poisson’s ratio of the NS as a strain function. Since the Poisson’s ratio is a property reserved for isotropic solids, we adopted a Poisson’s ratio defined as [68,69]

$$\nu \left(\epsilon \right)=-\frac{R_{\epsilon }-R_{i}}{\epsilon R_{i}}\;,$$

where the subindex ϵ and *i* refers to the radii and size in *z* at a given strain ϵ, and to radii and height at the beginning of the simulation (ϵ=0).

**Figure 5:**
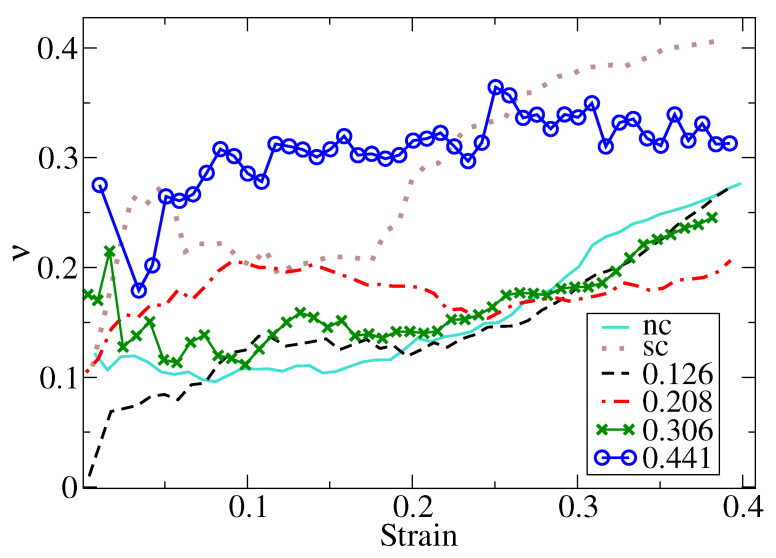
Effective Poisson’s ratio as a strain function for different ϕ values.

Figure 5 shows that Poisson’s ratio increased, stayed constant, and grew again with the strain. This behavior could be attributed to the grain boundary sliding of grains below the indenter and at the equatorial region of the nanosphere. Additionally, Poisson’s ratio of ϕ=0.441 was significantly higher than that of the other porous NS. A possible explanation is a deformation mediated by grain boundary sliding, which is activated at low strain values. ν values for single-crystal bulk Au with the Johnson et al. potential was reported to be 0.42 [67], in agreement with experimental evidence, which is higher than that reported here. We attribute this behavior to the finite size effect of the nanoparticle, which can suppress the lateral strain with other mechanisms, such as surface buckling or grain boundary sliding of the grains in contact with the indenter. Interestingly, our calculations of an effective Poisson’s ratio follows a similar behavior to the observed for copper nanorods where ν grows abruptly at low strains, achieves a plateau, and grows again for larger strains values [68].

### 3.3. Plastic Deformation Mechanism

**Figure 6:**
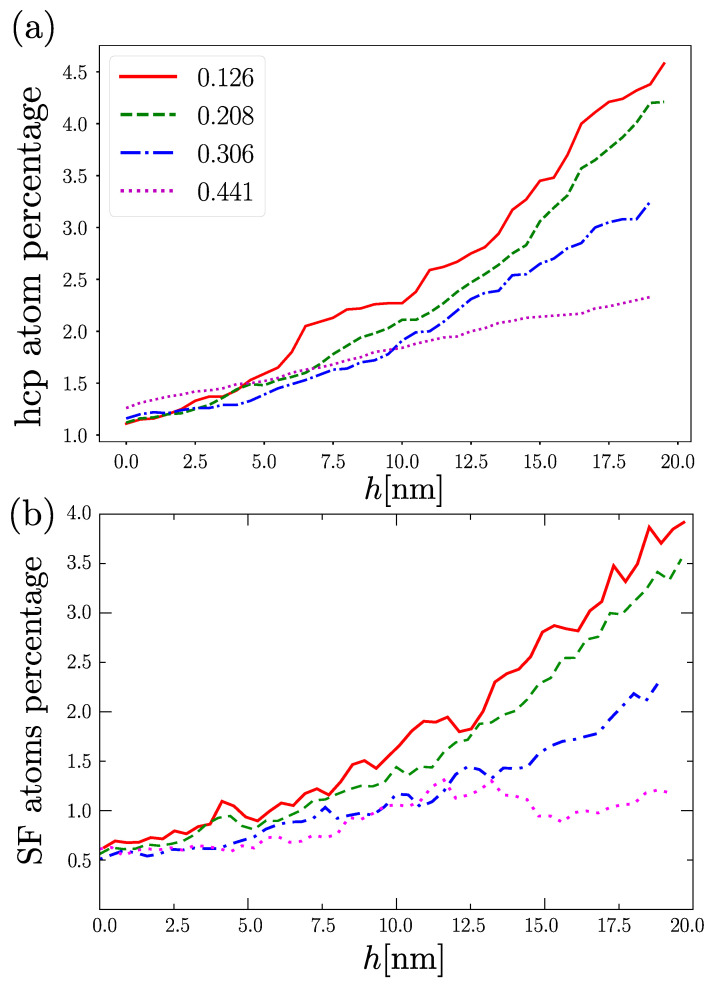
(**a**) % of hcp atoms as function of the indentation depth for ϕ=0.126,0.208,0.306,0.441. (**b**) % of atoms belonging to SF as function of the indentation depth.

Plastic deformations are characterized by multiple dislocation slips events taking place from grain boundary and filament structure, as was previously observed in Figure 1b. Since we studied an fcc porous nanostructure, quantitative analysis of slip planes (as twin boundaries and SF) can be included in hcp atoms to count as a strain function, depicted in Figure 6a. As expected, the number of hcp atoms increases exponentially with the strain, mainly due to the triggering of partial dislocations. Interestingly, the concentration of hcp atoms decreased when the system porosity increased. A possible explanation is that the large porosity and the grain boundary structure allowed for a ductile transition, which assisted in nanostructure deformation through filament bending and grain boundary sliding. A detailed analysis of the hcp atoms is presented in Figure 6b, by means of the CAT tool. CAT algorithm reveals that most of the hcp belong to SF. The increase in atoms belonging to SF is consistent with the increase of hcp atoms as a function of the indentation depth. The % of atoms was close to the % of hcp atoms detected by cna. The difference in the two values was due to the fact that some hcp atoms identified by CNA were detected in the form of twin boundaries according to CAT analysis. Of course, the contribution of twin boundaries is just a fraction of the SF atoms observed during the nanoindentation process, suggesting that the plastic deformation by slip of planes is dominated by the formation of SFs.

**Figure 7:**
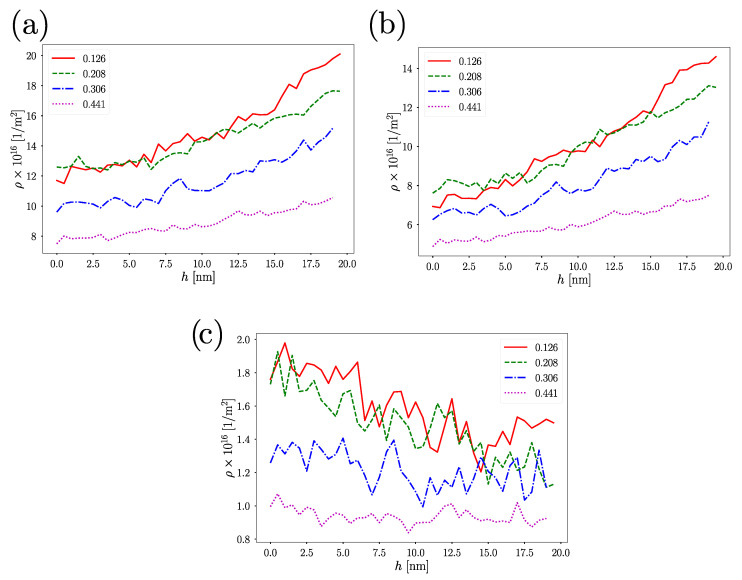
(**a**) Dislocation density as strain function. (**b**) Dislocation density considering only Shockley partial dislocation as a strain function. (**c**) Contribution of sessile dislocations to the dislocation density.

Dislocation analysis using the DXA algorithm (Figure 7a,b) shows dislocation densities increasing with the indentation depth, where the smaller the porosity was, the higher the dislocation density of the nanostructure. The dislocation densities observed here were comparable to those observed under uniaxial loading with strain rates of 10${}^{8}$ s${}^{-1}$, and also with the nanoindentation with indenters velocities close to 1.0–2.0 m/s [55]. Dissecting the dislocation density, we observed that dislocation activity was mainly composed of Shockley partial dislocation. SF are commonly a trace of 1/6<112> partial dislocation, in line with the increase in hcp atoms with the strain function observed in Figure 6. Surprisingly, for ϕ=0.441, both hcp and dislocation activity increased slowly with strain, suggesting that plastic deformation by linear defects was not the only mechanism influencing its mechanical response. Figure 7c shows sessile dislocation, such as stair-rod and hirt partial dislocations. Their contribution to dislocation density is smaller than a 5% of the total number of dislocations present in the sample, suggesting that hardening mediated by dislocation pinning as occurs in single-crystal hollow and non-hollow nanoparticles [6,20] should be ruled out. Typically, the high specific area of nanomaterials favors the migration and the annihilation of dislocations traveling in the crystal lattice of the nanomaterial. However, we observed a continuous accumulation of dislocations as the indenter reached higher depths. Interestingly, the inspection of the ϕ=0.441 NS revealed that dislocations that we detected only in the grain boundary structure of the NS, without evidence of dislocations inside the crystalline structure. The only trace of dislocation activity is the presence of SF suggesting that dislocations are nucleated, slip and are absorbed by the NS surface.

**Figure 8:**
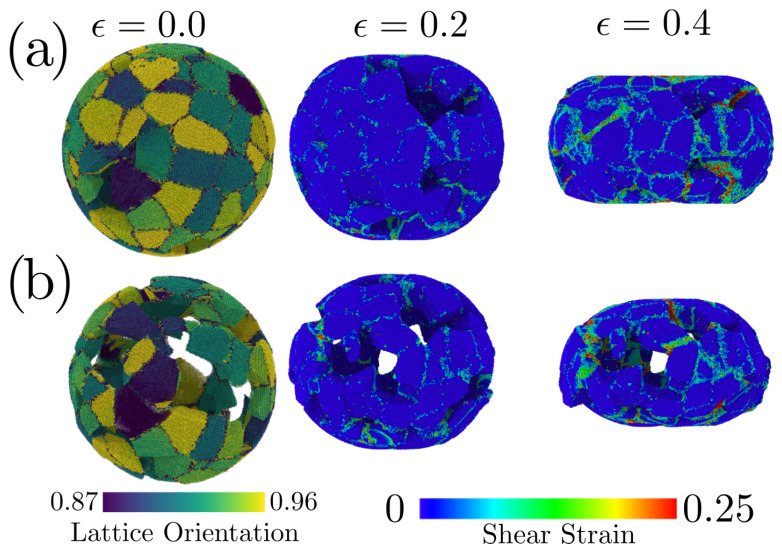
(**a**) Shear strain of a porous NS with ϕ=0.11. (**b**) Shear strain of porous NS of ϕ=0.403. To depict the grain boundary structure, we use as refence the case of ϵ=0.0, where both NS are colored according to their lattice orientation, calculated from the PTM algorithm.

By studying the shear strain in the whole sample, we understand the failure modes in this system due to shear accumulation in preferential zone of the nanostructure. As is depicted in Figure 8a, the ϕ=0.126 case showed shear strain localized in the contact zone in the grain boundary structure, leading to multiple strained zones located on grain boundaries or in the NS pores. For ϕ=0.441, the scenario is quite different since the shear strain at ϵ=0.208 is located in the contact zone and appears as a turned-on region in multiple pores of the NS. More extensive strain origin extended shear zones along the grain boundary structure to drive the grain boundary slip as shown for ϵ=0.441.

The large porosity of ϕ=0.441 NS reduced the grain boundary triple junction quantity. Then, when the NP was compressed, the porosity led to grain boundaries sliding. In this way, stress accumulation did not occur in grain boundaries or the lattice, avoiding a plastic deformation dominated by dislocation nucleation. A grain boundary sliding mechanism was consistent with the extended linear regime in the stress–strain curve, the lower dislocation density, and the small hcp concentration observed for ϕ=0.441.

## 4. Conclusions

The mechanical behavior of porous nanoshells (NS) was studied by means of molecular dynamics simulations of NS with different porosities. Our conclusions are:

- Porous NS displayed mechanical properties that were dependent on the porosity of the shell. For smaller ϕ values, a deformation dominated that was closely related to that observed for polycrystalline or single-crystalline NS; larger porosities render plastic deformation that is mediated by grain boundary sliding instead of dislocation activity.
- Young’s modulus decreased with the increase in the porosity of the nanoshell, as expected from the theories of porous materials. The Reissner model for thin shells provided the best fit for Young’s modulus determination. However, there was no quantitative agreement between the models to obtain the Young’s modulus of porous NS.
- Contact pressure was independent of nanoshell porosity, not revealing evidence of Hall–Petch hardening due to the grain boundaries or the filament structure, as is reported for nanoporous materials. Instead, the difference in load for the indented specimens increase inversely with the atomic contact area leading a constant contact pressure, which held for strains as high as 0.4.
- For low porosities, plastic deformation is mainly attributed to Shockley partial dislocations, with a minor contribution of sessile dislocations as stair-rods and Hirth locks. Dislocation densities values for ϕ>0.306 were smaller than those of other fcc nanoporous materials [32,55]. Nanoshells with high values of porosities (up to 0.441), show lower dislocation densities of almost an order of magnitude and a significant contribution of grain boundary sliding to deformation.

Due to their unique catalytic activity, there are numerous studies reporting synthesis techniques to control the porosity and size of porous nanoshells. Here, we show that porous NS also display an interesting combination of strength and ductility, which can be tailored by controlling the sample porosity in a similar way than conventional nanoporous materials [55,70]. Our work was limited to a fixed NS thickness with the goal of understanding the role played by the porosity. However, there are several studies that examine the role displayed by the shell thickness, and its contribution on NS hardness and flexibility [6,20,24]. Porous NS, due to their adjustable geometrical parameters such as NP size, shell thickness, and porous nanostructure, are an interesting mechanical material with wide room to tailor their mechanical properties.

## Data Availability

All data can be provided by the authors by request.

## Abbreviations

The following abbreviations are used in this manuscript:

NS	Nanoshell
sc	Single crystalline
pc	Polycrystalline
